# Evaluation of Antibiotic Resistance Genes in Commensal Gut Flora Among Healthy Individuals: A Hidden Reservoir for Resistance Transmission

**DOI:** 10.7759/cureus.97542

**Published:** 2025-11-23

**Authors:** Mansi Gupta, Dipak Patanvadia, Rachana A Bhavsar, Vijay S Rajak

**Affiliations:** 1 Microbiology, Bundelkhand Medical College, Sagar, IND; 2 Microbiology, Zydus Medical College and Hospital, Dahod, IND; 3 Microbiology, Dr. N D Desai Faculty of Medical Science and Research, Dharmsinh Desai University, Nadiad, IND; 4 Microbiology, Government Medical College, Datia, Datia, IND

**Keywords:** antibiotic resistance genes, antimicrobial resistance, commensal bacteria, gut microbiota, healthy adults, mobile genetic elements

## Abstract

Background

Antimicrobial resistance (AMR) poses a major global health challenge, undermining the effectiveness of existing antibiotics and complicating the management of infectious diseases. The human gut microbiome serves as an important reservoir of antibiotic resistance genes (ARGs), which can be transferred among bacterial populations, including those inhabiting healthy individuals. Understanding the diversity and distribution of these ARGs at the community level is essential to identifying the hidden reservoirs of resistance within apparently healthy populations. However, data on the prevalence and determinants of ARGs in the general population of India remain limited.

Methods

A community-based cross-sectional study was conducted among 150 healthy adults (aged 18-60 years) in a tertiary care center of Central India from January to September 2025. Stool samples were analyzed using culture and multiplex quantitative PCR for nine major ARGs (blaTEM, blaCTX-M, blaNDM, tetM, ermB, sul1, qnrS, vanA, and mcr-1) and mobile genetic elements (MGEs). Associations between ARG carriage and demographic and exposure factors were assessed using the chi-square, Kruskal-Wallis, and regression analyses.

Results

The most common ARGs were tetM (42.7%), blaTEM (38.7%), and sul1 (34%) genes. ARG richness was significantly associated with recent antibiotic use (χ² = 17.3, p < 0.001) and MGE detection (χ² = 12.5, p < 0.001). Probiotic use was independently protective against blaTEM carriage (adjusted odds ratio = 0.19, 95% CI = 0.05-0.69, p = 0.011), whereas MGE detection showed a positive trend (p = 0.060). Linear regression (R² = 0.283) indicated that younger age (p = 0.014) and “Other” sex (p < 0.001) were associated with a higher total ARG load.

Conclusion

Healthy individuals harbor diverse and transmissible ARGs in their gut microbiota. Antibiotic exposure and MGEs increase ARG diversity, whereas probiotics may reduce blaTEM carriage. These findings highlight the need for One Health surveillance and prudent antimicrobial stewardship to limit the spread of AMR at the community level.

## Introduction

Antimicrobial resistance (AMR) has become one of the most formidable challenges to global health, undermining the efficacy of antibiotics and threatening the successful treatment of infectious diseases [1]. The human gut microbiome, a complex and dynamic ecosystem comprising trillions of microorganisms, is increasingly recognized as a significant reservoir of antibiotic resistance genes (ARGs) that contribute to the overall burden of resistance [2]. These ARGs, collectively referred to as the gut resistome, are not limited to pathogenic bacteria and are also present in commensal species that inhabit the intestinal tract. Through mechanisms such as horizontal gene transfer (HGT), these commensal bacteria can disseminate resistance determinants to potential pathogens, thereby facilitating the spread of AMR within both community and healthcare settings [3,4].

Recent metagenomic studies have highlighted the widespread occurrence of diverse ARGs even among clinically healthy individuals. In a descriptive pilot study conducted in Australia, ARGs were detected in all fecal samples from healthy women and infants, with 64 unique genes conferring resistance to 12 antibiotic classes, most commonly tetracyclines, β-lactams, and macrolide-lincosamide-streptogramin B (MLSB) antibiotics [1]. Similarly, large-scale analyses of gut microbiomes from healthy and diseased populations have demonstrated that resistome composition is influenced by disease status, geographic location, and antibiotic consumption patterns [2,3]. The use of antibiotics at the population level has been shown to strongly correlate with the prevalence and diversity of ARGs, indicating that widespread antimicrobial exposure shapes resistome profiles [2]. Studies utilizing advanced molecular techniques, such as bacterial Hi-C sequencing, have further demonstrated the frequent occurrence of HGT among gut microorganisms, mediated by mobile genetic elements (MGEs), including plasmids, integrons, and transposons [4]. Beyond systemic antibiotic exposure, clinical and dental interventions can also alter the composition and function of oral and gut microbiota, further illustrating how medical practices influence human-associated resistomes [5-7].

The accumulation of resistance genes over time, particularly those conferring tetracycline and efflux-related resistance, has been observed in older individuals, reflecting age-dependent enrichment of ARGs within the gut microbiota [8]. Longitudinal analyses of healthy adults have shown that while the abundance of certain ARGs fluctuates, the acquisition of novel resistance determinants, such as those associated with international travel, remains rare without external exposure [9]. Moreover, the increasing use of probiotic supplements, some of which contain transferable resistance genes, poses additional concerns regarding their contribution to the expansion of the gut resistome [10]. At the same time, several clinical and experimental studies suggest that specific probiotic formulations may modulate gut microbial communities in ways that could limit colonization by resistant strains. However, these effects are strain-, host-, and context-dependent [10,11]. Collectively, these findings emphasize that the human gut microbiome in healthy individuals acts as a “hidden reservoir” for clinically relevant resistance genes that can potentially be transmitted to pathogenic bacteria [11].

Despite these significant insights from developed regions, limited data exist from low- and middle-income countries, such as India, where antibiotic misuse, over-the-counter availability, and environmental contamination are common. Understanding the baseline prevalence and diversity of ARGs in the gut flora of healthy individuals is critical for identifying the community reservoir of resistance and the factors influencing its distribution. Therefore, the present study aimed to evaluate the prevalence and diversity of ARGs in the commensal gut flora of healthy individuals and to determine the associations between ARG carriage and demographic, environmental, and behavioral factors, such as antibiotic exposure, diet, and animal contact. This study also sought to identify the occurrence of MGEs associated with ARGs to assess the potential for HGT. In such settings, community-based data on ARGs and MGEs are crucial for determining the potential for HGT and the silent dissemination of resistance outside hospital environments. Therefore, the primary objective of the present study was to evaluate the prevalence and diversity of ARGs in the commensal gut flora of healthy individuals in Central India. The secondary objectives were to determine the associations between ARG carriage and demographic, environmental, and behavioral factors (including recent antibiotic and probiotic exposure, diet type, animal contact, and travel history) and to identify the occurrence of MGEs associated with ARGs as indicators of HGT potential in the community. We hypothesized that recent antibiotic exposure and MGE detection would be related to increased ARG richness. In contrast, the use of probiotics would be associated with a lower prevalence of selected ARGs, particularly β-lactam resistance determinants. By characterizing the gut resistome in healthy individuals, this study will provide essential baseline data to support antibiotic stewardship initiatives and inform public health strategies to mitigate AMR transmission in the community.

## Materials and methods

Study design and setting

This community-based, cross-sectional, observational study was conducted in the Department of Microbiology at the Government Medical College, Datia, from January to September 2025. All laboratory work was conducted in a biosafety level 2 (BSL-2) facility equipped for microbiological and molecular analysis.

Study population and sampling

Participants were recruited through community outreach activities and hospital health check-up camps. Purposive sampling was used to ensure inclusion of both rural and urban residents across gender and age strata. This non-probability method was chosen for feasibility; however, it may limit generalizability.

Inclusion Criteria

Healthy adults aged 18-60 years without chronic illness or current medication were included in the study.

Exclusion Criteria

Individuals with current acute gastrointestinal illness, recent hospitalization for any infection within the past three months, or any known immunocompromising condition (e.g., HIV infection, chemotherapy, or long-term immunosuppressive therapy) were excluded. Recent antibiotic or probiotic use was not an exclusion criterion and was instead recorded as an exposure variable for analysis.

To improve reproducibility, we specify that during the study period, 210 adults were approached, 174 met the eligibility criteria, and 150 provided stool samples and were included in the final analysis.

Data collection and laboratory procedure

Data Collection and Sample Handling

Data were collected using a semi-structured questionnaire that recorded demographic characteristics, recent antibiotic or probiotic use, diet type, animal contact, and travel history. Fresh stool samples were collected in sterile screw-capped containers, transported to the laboratory within six hours under refrigerated conditions (4-8°C), and processed immediately upon arrival. Samples were divided into two aliquots: one for culture-based identification and the other for molecular analysis.

Culture and Phenotypic Analysis

Conventional culture was performed on MacConkey agar and bile esculin agar to isolate commensal *Enterobacterales* and *Enterococcus* species. Bacterial identification was carried out using standard biochemical tests, and antimicrobial susceptibility testing was performed according to the Clinical and Laboratory Standards Institute (CLSI) 2023 guidelines.

Genomic DNA Extraction

Genomic DNA was extracted from stool samples using the QIAamp DNA Stool Mini Kit (Qiagen, Hilden, Germany) following the manufacturer’s protocol. DNA purity and concentration were assessed spectrophotometrically, ensuring an A260/A280 ratio between 1.8 and 2.0 to confirm nucleic acid quality before downstream molecular assays.

Quantitative PCR Detection of Antibiotic Resistance Genes

Detection of major ARGs was conducted using multiplex quantitative PCR (qPCR) to target nine commonly encountered genes representing key resistance mechanisms: blaTEM (penicillin resistance), blaCTX-M (extended-spectrum β-lactamase), blaNDM (carbapenem resistance), tetM (tetracycline resistance), ermB (macrolide resistance), sul1 (sulfonamide resistance), qnrS (quinolone resistance), vanA (glycopeptide resistance), and mcr-1 (colistin resistance).

Primer Design and Amplification Conditions

Primers were designed from publicly available sequences in the National Center for Biotechnology Information (NCBI) GenBank database and validated through BLAST analysis to ensure specificity. Each 25 µL qPCR reaction mixture contained 12.5 µL of 2× SYBR Green Master Mix (Applied Biosystems, Waltham, MA), 0.4 µM of each primer, and 2 µL of extracted DNA template. Amplification was performed on a StepOnePlus Real-Time PCR System (Applied Biosystems) using the following cycling conditions: initial denaturation at 95°C for five minutes, followed by 40 cycles of denaturation at 95°C for 15 seconds, annealing at 55-58°C for 30 seconds (depending on the target gene), and extension at 72°C for 30 seconds.

The 16S rRNA gene was used as an internal control to normalize bacterial load across all samples. Each run included a no-template control (NTC) to check for contamination and a positive control strain for each gene (e.g., *E. coli* ATCC 35218 for blaTEM, *Enterococcus faecium* vanA-positive control for vanA, and *E. coli* NDM-1 for blaNDM). Specificity was confirmed by melting curve analysis, showing single, sharp peaks for each target. Only reactions with consistent cycle threshold (Ct) values (≤0.5 cycle difference between replicates) were considered valid.

Standard curves generated from 10-fold serial dilutions of positive control DNA yielded amplification efficiencies between 90% and 110% and R² ≥ 0.98 for all targets. Limits of detection (LOD) ranged from X-Y copies/reaction (details in the Appendices).

Detection of MGEs

To evaluate the potential for horizontal gene transfer, class I and II integron integrase genes (intI1 and intI2) were amplified by conventional PCR. Amplified products were verified through Sanger sequencing to confirm sequence identity. The presence of MGEs was analyzed in relation to ARG profiles to assess possible co-occurrence and transmission potential.

Metagenomic Validation and Quality Control

To validate the qPCR findings and ensure data robustness, a subset of 30 stool samples (20% of the total) was randomly selected for shotgun metagenomic sequencing using the Illumina MiSeq platform (Illumina, Inc., San Diego, CA). Samples were stratified by age and sex before selection. Sequencing reads were quality-filtered (Q ≥ 30) and mapped to the Comprehensive Antibiotic Resistance Database (CARD v4.0) and ResFinder 2024 databases using thresholds of ≥90% identity and ≥80% coverage. Cross-validation of metagenomic and qPCR data for corresponding samples demonstrated >95% concordance for all major ARGs, confirming high analytical accuracy and reproducibility.

Data Validation and Reproducibility

All assays were conducted in triplicate to ensure reliability. Negative extraction controls were included in each DNA isolation batch to detect potential contamination. In addition, 10% of the total samples were randomly reanalyzed to assess inter-assay reproducibility. These internal quality control measures verified the precision, stability, and reproducibility of both culture-based and molecular assays.

Statistical analysis

Data were analyzed using Jamovi 2.3.28. Descriptive statistics summarized variables as mean ± SD, median (IQR), or frequency (%). Normality was tested by the Shapiro-Wilk test. Group differences were assessed using the Mann-Whitney U, Kruskal-Wallis H, and chi-square tests, with effect sizes (ε²) reported. Binary logistic regression identified predictors of specific ARG carriage, and multiple linear regression examined determinants of total ARG abundance. Model fit was evaluated using Akaike information criterion (AIC), R², and McFadden’s R². All tests were two-tailed with significance set at p < 0.05.

All data entries were reviewed for completeness before analysis. Variables with missing or incomplete responses were cross-verified against source records. As the final dataset showed no missing observations for key demographic or laboratory variables, complete-case analysis was applied.

Ethical considerations

Written informed consent was obtained from all participants after explaining the study purpose, risks, and rights to them. Confidentiality was maintained by anonymized coding of samples and restricted data access. All samples were handled under BSL-2 conditions, and biomedical waste was managed by the Government of India Biomedical Waste Management Rules (2016, amended 2023). Ethical approval was obtained from the Institutional Ethics Committee of Biomedical and Health Research in Human Participants (IECBMHR), Government Medical College, Datia (Approval No.: 125/Micro/GMC/IECBMHR/2024).

## Results

A total of 150 clinically healthy participants were enrolled in this study. The mean age was 39.0 ± 12.6 years, and the mean body mass index (BMI) was 24.1 ± 3.8 kg/m². Males comprised 52% of the study population, and approximately 60% of the participants resided in urban areas. Among all participants, 35% reported antibiotic use within the past three months, 28% had consumed probiotics, and 32% had direct animal contact. All 150 participants had completed the questionnaire and laboratory testing and were included in the final analyses, with recent antibiotic and probiotic use treated as exposure variables rather than exclusion criteria.

ARG presence and distribution

**Figure 1:**
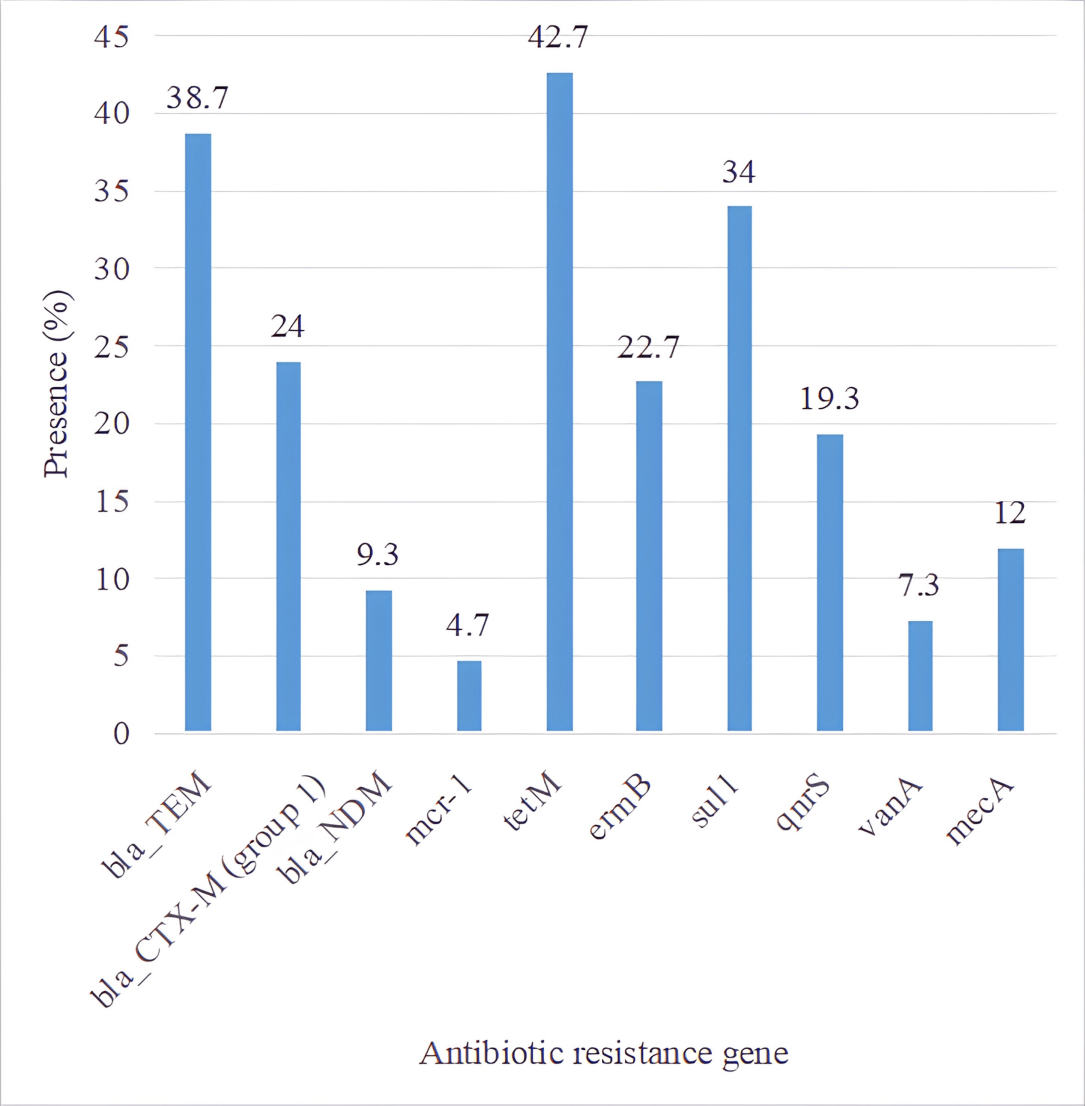
Distribution of antibiotic resistance genes among bacterial isolates. This bar graph shows the distribution of key antibiotic resistance genes detected among commensal bacterial isolates. The tetM gene (42.7%) was the most prevalent, followed by blaTEM (38.7%) and sul1 (34%). Genes conferring β-lactam resistance (blaCTX-M, blaTEM, blaNDM) and tetracycline resistance (tetM) were most frequently detected, indicating widespread community-level exposure to these antibiotic classes. Data are expressed as percentages of total isolates carrying each gene.

The most prevalent genes were tetM (42.7%), blaTEM (38.7%), and sul1 (34%), followed by blaCTX-M group 1 (24%), ermB (22.7%), and qnrS (19.3%). Moderate frequencies were observed for the mecA (12%), blaNDM (9.3%), vanA (7.3%), and mcr-1 (4.7%) genes (Figure 1).

The predominance of tetracycline, β-lactam, and sulfonamide resistance genes suggests that environmental and community-level exposure to these antimicrobial classes contributes to their persistence, even in healthy individuals.

Association of exposure factors with ARG richness

ARG diversity (richness) was significantly associated with antibiotic exposure and the presence of MGEs. Participants with recent antibiotic use exhibited higher ARG richness (χ² = 17.3, p < 0.001) than non-users, and MGE detection was also linked to increased ARG diversity (χ² = 12.5, p < 0.001). Sex and occupation showed no significant association (p > 0.05), indicating that microbial exchange dynamics and environmental exposure were stronger determinants of ARG variation than demographic factors (Table 1).

**Table 1:** Association between selected variables and antimicrobial resistance. This table summarizes the relationship between exposure factors (recent antibiotic use, mobile genetic elements, sex, and occupation) and ARG richness, defined as the number of distinct resistance genes detected per sample. The Kruskal–Wallis H test was used to assess group differences, with effect sizes (ε²) provided. *** P < 0.001 - significant; NS: not significant (p > 0.05). ARG: antibiotic resistance gene; MGE: mobile genetic element.

Variable (grouping factor)	χ² (H)	p-value	ε² (Effect size)
Recent antibiotic use	17.3	<0.001***	0.116
MGE detection	12.5	<0.001***	0.0837
Sex	0.522	0.77^NS^	0.0035
Occupation	6.58	0.16^NS^	0.0442

**Figure 2:**
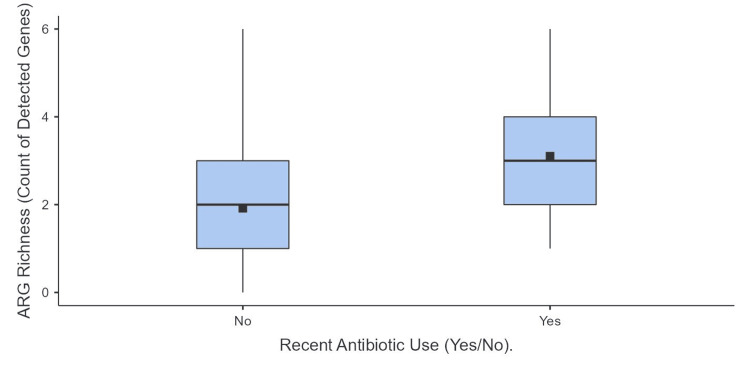
Association between recent antibiotic use and ARG richness. Boxplot depicting antibiotic resistance gene (ARG) richness among participants with and without recent antibiotic use. ARG richness represents the count of distinct resistance genes detected per individual. Boxes indicate the interquartile range (IQR), the central line shows the median, and whiskers represent 1.5 × IQR. Filled dots denote individual data points. Individuals with recent antibiotic use exhibited higher median ARG richness, suggesting selection pressure from antimicrobial exposure.

Figure 2 shows that participants with recent antibiotic exposure demonstrated a higher median ARG richness and wider interquartile range than those without, supporting that antimicrobial exposure is associated with a higher diversity of resistance determinants in the gut microbiota, although causality cannot be inferred from this cross-sectional analysis.

**Figure 3:**
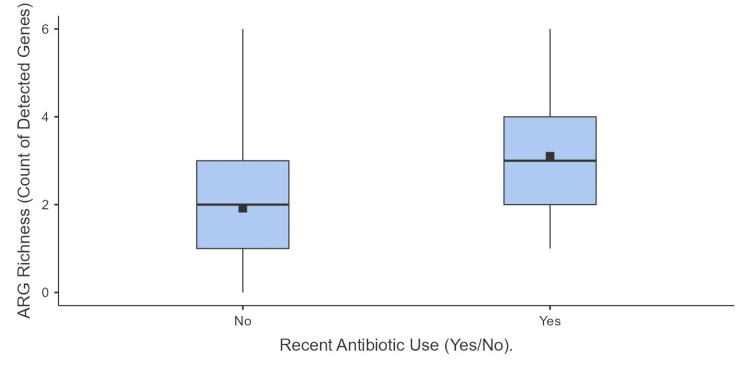
Association between mobile genetic elements and ARG richness. Boxplot illustrating the relationship between the detection of mobile genetic elements (MGEs) and antibiotic resistance gene (ARG) richness. ARG richness refers to the total number of unique resistance genes detected per sample. Participants with MGE-positive samples demonstrated higher ARG richness, suggesting enhanced potential for horizontal gene transfer. The central line denotes the median; box boundaries indicate IQR; whiskers represent 1.5 × IQR.

Similarly, Figure 3 illustrates that participants harboring MGEs had a greater ARG richness than MGE-negative individuals. The elevated median values highlight the role of horizontal gene transfer in facilitating ARG accumulation and spread among the commensal bacterial populations.

Phenotype-genotype relationships

Strong concordance was observed between the molecular and phenotypic resistance profiles. blaCTX-M was detected in 31.0% of the isolates and was significantly associated with third-generation cephalosporin resistance (χ² = 10.5, p = 0.001). Both blaNDM and mcr-1 were identified in 8.6% of the isolates and were strongly associated with carbapenem and colistin resistance, respectively (χ² = 24.0, p < 0.001) (Table 2). These findings validate that molecular screening accurately predicts phenotypic resistance outcomes and indicate that commensal flora may serve as reservoirs for clinically relevant resistance determinants.

**Table 2:** Association between phenotypic and genotypic resistance patterns. Associations were evaluated using the Pearson chi-square (χ²) test. Data demonstrate strong genotype–phenotype agreement for β-lactam, carbapenem, and colistin resistance. P < 0.05 is considered statistically significant. ** p < 0.01; *** p < 0.001. bla: β-lactamase gene; NDM: New Delhi metallo-β-lactamase; mcr-1: colistin resistance gene.

Phenotype–genotype pair	Phenotype: Resistant (n)	Phenotype: Sensitive (n)	χ²	p-value
Third-generation cephalosporins vs. bla CTX-M	21 (12 gene-positive)	37 (6 gene-positive)	10.5	0.001**
Carbapenems vs. bla NDM	4 (3 gene-positive)	54 (2 gene-positive)	24	<0.001***
Colistin vs. mcr-1	4 (3 gene-positive)	54 (2 gene-positive)	24	<0.001***

Determinants of blaTEM gene carriage

Binary logistic regression analysis identified probiotic consumption as a significant independent protective factor for blaTEM gene carriage. Participants who had consumed probiotics within the past three months were 80% less likely to harbor blaTEM-positive bacteria (adjusted odds ratio (AOR) = 0.19, 95% CI = 0.05-0.69, p = 0.011). Other variables, including antibiotic use, animal exposure, residence type, and MGE detection, were not statistically significant, although MGE detection showed a marginally positive trend (AOR = 2.10, p = 0.060) (Table 3). These regression findings indicate an inverse association between probiotic use and blaTEM carriage rather than a proven causal effect.

**Table 3:** Logistic regression analysis of predictors associated with blaTEM gene carriage. Binary logistic regression model identifying independent predictors of blaTEM gene carriage. Results are expressed as adjusted odds ratios (AOR) with 95% confidence intervals (CIs), standard errors (SE), Wald z-values, and p-values. Model fit was assessed using deviance, Akaike information criterion (AIC), and McFadden’s R². Model fit: Deviance = 186; AIC = 200; McFadden’s R² = 0.072, which explained approximately 7.2% of the variance in blaTEM gene carriage, indicating an acceptable overall fit. * P < 0.05 - significant; NS: not significant- p > 0.05. MGE: mobile genetic element.

Predictor	β (Estimate)	SE	Wald Z	Odds ratio (OR)	95% CI for OR (Lower–Upper)	p-value
Intercept	−0.3355	0.379	−0.886	0.72	0.34 – 1.50	0.376^NS^
Recent antibiotic use (Yes vs. No)	−0.0820	0.457	−0.179	0.92	0.38 – 2.26	0.858^NS^
Animal contact (Yes vs. No)	0.3327	0.36	0.925	1.4	0.69 – 2.82	0.355^NS^
Probiotic use (Yes vs. No)	−1.6741	0.661	−2.531	0.19	0.05 – 0.69	0.011*
Recent travel (Yes vs. No)	−0.5077	0.38	−1.338	0.6	0.29 – 1.27	0.181^NS^
Residence (Urban vs. Rural)	−0.1759	0.361	−0.488	0.84	0.41 – 1.70	0.626^NS^
Mobile genetic elements (MGE detected vs. not detected)	0.7431	0.395	1.881	2.1	0.97 – 4.56	0.06^NS^

These findings are consistent with the possibility that probiotic-associated modulation of the gut microbiota may influence colonization by blaTEM-positive commensals; however, the modest model fit and the cross-sectional design mean that residual confounding cannot be excluded, and causality cannot be established.

Determinants of total ARG load

Multiple linear regression analysis (R² = 0.283) revealed that age and sex significantly influenced the total ARG copy number normalized to the 16S rRNA gene. Each additional year of age corresponded to a reduction of approximately 146 ARG copies (p = 0.014), indicating a higher resistome load in younger individuals. Participants identifying as “Other” sex displayed markedly higher ARG copy numbers than females (p < 0.001), whereas males showed no significant difference (p = 0.140) (Table 4).

**Table 4:** Multiple linear regression predicting total ARG copies per 16S rRNA gene. Multiple linear regression model showing demographic and biological predictors of total ARG abundance, expressed as ARG copy number normalized to the 16S rRNA gene. Results include regression coefficients (β), standard errors (SE), t statistics, and p-values. Model goodness-of-fit was evaluated using R and R² values. Model fit: R = 0.532, R² = 0.283, which explained approximately 28.3% of the variance in total ARG copies per 16S rRNA gene. * p < 0.05, ** p < 0.01, and *** p < 0.001; NS = not significant (p > 0.05). ARG: antibiotic resistance gene; MGE: mobile genetic element; rRNA: ribosomal ribonucleic acid.

Predictor	β (Estimate)	SE	t	p-value
Intercept	16,161	5,177.00	3.122	0.002**
Sex (Male vs. Female)	2,200	1,483.20	1.483	0.14^NS^
Sex (Other vs. Female)	58,440	9,104.40	6.419	<0.001***
Recent antibiotic use (Yes vs. No)	−1,427	1,872.40	−0.762	0.447^NS^
BMI (per unit increase)	−232	193.5	−1.200	0.232^NS^
Age (per year increase)	−146	58.7	−2.491	0.014*
MGE detected (Yes vs. No)	184	1,671.00	0.11	0.912^NS^

BMI, antibiotic use, and MGE were not significant predictors (p > 0.05). These findings suggest that intrinsic biological characteristics, particularly age and sex, may exert a greater influence on resistome abundance than short-term antibiotic or environmental exposures in this cohort.

## Discussion

This study reveals that ARGs are prevalent among healthy individuals, indicating the silent spread of AMR within the community. We identified several resistance genes, including tetM (42.7%), blaTEM (38.7%), and sul1 (34%), which align with results from population studies in Australia and other countries.

Guernier-Cambert et al. [1] found that healthy adults and infants in southeastern Australia carried 64 different ARGs that confer resistance to 12 types of antibiotics, including β-lactams, tetracyclines, and MLSB antibiotics. Similar to our results, these resistance genes were also found in individuals who had not taken antibiotics, suggesting that environmental factors and diet influence the gut resistome. On the other hand, Huang et al. found that ICU healthcare workers had significantly higher levels and types of ARGs than those in the community, indicating that hospital work contributes to the presence of ARGs in the gut [12]. Together, these studies demonstrate that both community and work settings can increase ARGs, even in the absence of active infection. In our study, we utilized self-reported recent antibiotic use among community-dwelling adults to examine real-world exposure and ARG richness. However, this approach has limitations due to potential recall and reporting bias.

Geography and lifestyle also influence the spread of resistance genes. Qiu et al. found that tetracycline and β-lactam resistance genes are the most prevalent, and that regional and antibiotic use patterns shape ARG distributions [3]. Like other large studies that link antibiotic use to the composition of resistance genes [2,3], we found that individuals who had recently used antibiotics had a greater number of ARGs (χ² = 17.3, p < 0.001). This supports the idea that antibiotic exposure is linked to an increased number of resistance genes in the community, although it does not prove a direct cause. Lebeaux et al. [13] also showed that infants who took antibiotics had more ARGs and changes in their gut bacteria, especially when they were in daycare.

We found a strong link between MGEs and the number of ARGs, which matches other research showing that MGEs help spread genes between bacteria. Tokuda et al. referred to MGEs as evolutionary tools that help bacteria adapt [14]. Shang et al. and Johansson et al. also found strong links between MGEs and ARGs in various environments, suggesting that gene movement is a global process [15,16]. Forster et al. demonstrated in experiments that harmless bacteria can transfer ARGs to harmful ones using MGEs that are active in many hosts [17]. Horne et al. found that MGEs can either increase or decrease gene transfer [18]. Our results are similar, indicating that gene movement facilitates the growth of ARGs in the gut bacteria of healthy individuals. However, because our study is cross-sectional, we should view MGEs as markers linked to more ARGs and possible gene transfer, rather than as definite causes of the patterns we observed.

The observed genotype-phenotype concordance for blaCTX-M, blaNDM, and mcr-1 validates molecular detection as a predictor of resistance patterns, supporting the findings of Almeida et al. and Stephens et al. that F-plasmid-mediated ARGs in commensal flora remain transmissible [19,20]. Crits-Christoph et al. further described the microbiome as a “hidden gene bank” for ARGs capable of horizontal transfer to pathogens [11]. Taken together, the high concordance between genotypic and phenotypic resistance in our data, combined with established evidence of ARG mobility in commensal communities [11,17,20], supports the biological plausibility that clinically healthy individuals can serve as reservoirs of transferable resistance determinants.

People who took probiotics appeared to be less likely to carry blaTEM (AOR = 0.19, p = 0.011). However, Radovanovic et al. and Tóth et al. warned that probiotics can also have ARGs that can be passed on [10,21]. Montassier et al. demonstrated that the effects of probiotics vary depending on the individual and whether they have taken antibiotics [22]. Other studies have found that certain probiotics or treatments that alter the microbiome can modify the bacteria in the mouth and gut, thereby reducing signs of imbalance [5,6]. This provides a reason why using certain probiotics may lower the likelihood of carrying blaTEM. Our results align with the notion that probiotics may help prevent certain β-lactam-resistant bacteria from growing in the gut. Still, because our model only explained a small part of the difference and due to possible confounding factors and the cross-sectional design, we should view this as an association, rather than proof that probiotics protect against blaTEM.

Age and sex were significant predictors of total ARG load, with younger individuals showing higher ARG copy numbers. Similar patterns were observed by Van Gompel et al., who linked biological and occupational factors to the resistome variability [23]. Pan et al. associated gut ARG profiles with the risk of chronic disease [24]. In our cohort, higher ARG abundance in younger adults and in participants identifying as “Other” sex suggests that intrinsic host characteristics, social contact networks, and unmeasured behavioral factors may interact to shape the resistome independently of short-term antibiotic exposure. However, these mechanisms could not be disentangled within the present study.

One main strength of this study is that we combined traditional culture and resistance testing with multiplex quantitative PCR and some metagenomic sequencing. This provided us with a detailed understanding of resistance from both functional and genetic perspectives. We employed internal controls, conducted tests in triplicate, and rechecked some samples to enhance the reliability of our results. By sampling people from both rural and urban areas and collecting detailed information on antibiotics, probiotics, diet, and animal contact, we created valid baseline data for AMR monitoring in a low- and middle-income setting.

Limitations

Using purposive sampling in this study may have introduced selection bias, making it more challenging to generalize the results to the entire community. While multiplex qPCR helped us detect significant resistance genes, it did not cover all possible ARGs. We only did metagenomic sequencing on some samples, which may have limited our comparisons. Information about antibiotic and probiotic use was self-reported, which may have introduced recall bias. Additionally, since we considered recent antibiotic and probiotic use as exposure factors, reporting errors could have impacted our results. Because the study is cross-sectional, we cannot say that exposures cause resistance patterns. Therefore, all links between exposures (such as antibiotics, probiotics, MGEs, and demographic factors) and ARGs in this study should be viewed as associations rather than causal relationships. Future studies involving more centers, larger sample sizes, and additional gene testing are needed to confirm and build upon these findings.

## Conclusions

This study demonstrated that clinically healthy individuals harbor diverse ARGs, particularly those conferring resistance to tetracyclines, β-lactams, and sulfonamides. Recent antibiotic exposure and the presence of MGEs were key drivers of increased ARG richness, whereas probiotic use appeared to be protective against blaTEM carriage. Age and sex also influenced the total ARG abundance. These findings highlight that antimicrobial resistance extends beyond clinical settings, emphasizing the need for integrated One Health surveillance, prudent antibiotic stewardship, and regulation of probiotic use to mitigate silent, community-level dissemination of resistance determinants.

## Appendices

**Table 5:** qPCR assay performance characteristics for nine target antibiotic resistance genes (ARGs). Efficiencies between 90% and 110% indicate optimal quantitative PCR (qPCR) performance. R² values ≥ 0.99 reflect strong linearity of standard curves. Limits of detection (LOD) were determined using 10-fold serial dilutions of validated positive control DNA. All primer sets showed single melting-curve peaks, indicating high specificity.

ARG target	Amplicon size (bp)	Efficiency (%)	R²	LOD (copies/reaction)	Dynamic range (log10)
blaTEM	108	98.4	0.998	20	10⁶–10¹
blaCTX-M	122	93.7	0.996	25	10⁶–10¹
blaNDM	101	94.5	0.995	15	10⁶–10¹
tetM	164	102.1	0.997	10	10⁶–10¹
ermB	143	95.8	0.994	30	10⁶–10¹
sul1	118	97.2	0.995	20	10⁶–10¹
qnrS	123	90.6	0.992	40	10⁶–10¹
vanA	192	99.0	0.998	10	10⁶–10¹
mcr-1	159	91.3	0.993	25	10⁶–10¹
